# Roles of Hsp70s in Stress Responses of Microorganisms, Plants, and Animals

**DOI:** 10.1155/2015/510319

**Published:** 2015-11-16

**Authors:** Anmin Yu, Ping Li, Ting Tang, Jiancai Wang, Yuan Chen, Li Liu

**Affiliations:** ^1^Key Laboratory of Economic Plants and Biotechnology, Kunming Institute of Botany, Chinese Academy of Sciences, Kunming, Yunnan 650201, China; ^2^Yunnan Key Laboratory for Wild Plant Resources, Kunming, Yunnan 650201, China; ^3^Key Laboratory of Tropical Plant Resource and Sustainable Use, Xishuangbanna Tropical Botanical Garden, Chinese Academy of Sciences, Kunming, Yunnan 650223, China; ^4^Yangjiang Forestry Administration, Dali, Yunnan 672401, China

## Abstract

Hsp70s (heat shock protein 70s) are a class of molecular chaperones that are highly conserved and ubiquitous in organisms ranging from microorganisms to plants and humans. Most research on Hsp70s has focused on the mechanisms of their functions as molecular chaperones, but recently, studies on stress responses are coming to the forefront. Hsp70s play key roles in cellular development and protecting living organisms from environmental stresses such as heat, drought, salinity, acidity, and cold. Moreover, functions of human Hsp70s are related to diseases including neurological disorders, cancer, and virus infection. In this review, we provide an overview of the specific roles of Hsp70s in response to stress, particularly abiotic stress, in all living organisms.

## 1. Introduction

Proteins fold into the correct three-dimensional structures with the assistance of chaperone proteins to avoid abnormal folding and aggregation [1]. Heat shock proteins (Hsps), molecular chaperones originally discovered based on their upregulation in response to heat stress, have been divided into five different families based on molecular weight: Hsp100, Hsp90, Hsp70, Hsp60, and small (s) Hsp [2]. Living organisms must adapt to temperature changes during their lifetime, and the Hsp70s are found in every kingdom. For instance, photosynthetic eukaryotes contain four types of Hsp70s which localize to different cellular compartments: cytoplasm, mitochondrion (MT), chloroplast (CP), and endoplasmic reticulum (ER) [3]. Hsp70s are involved in many cellular processes, including protein folding, protein translocation across membranes, and regulation of protein degradation. This review will focus on types and numbers of Hsp70s in various kingdoms species, as well as their functions in stress responses.

## 2. Structure and Isotype of Hsp70s

**Figure 1:**
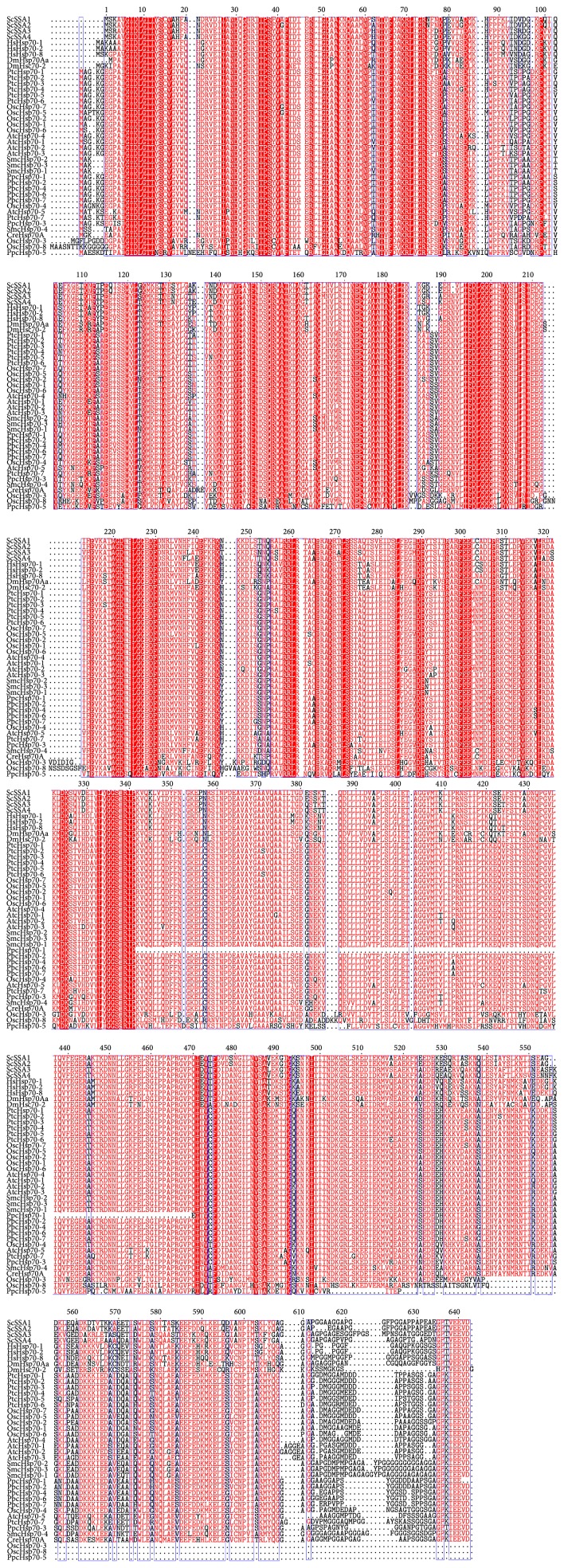
Analysis of the conserved domains of partial Hsp70s in cytoplasm group from various kingdoms. The Hsp70 members from* Saccharomyces cerevisiae* (Sc),* Homo sapiens* (Hs),* Drosophila melanogaster* (Dm),* Chlamydomonas reinhardtii* (Cre),* Physcomitrella patens* (Pp),* Selaginella moellendorffii* (Sm),* Oryza sativa* (Os),* Arabidopsis thaliana* (At), and* Populus trichocarpa* (Pt).

Hsps are pivotal molecular chaperones that function in response to proteotoxic stress by refolding misfolded proteins. Hsp70 (called DnaK in prokaryotes) is a 70-kDa protein that represents the best-known heat shock protein [4]. Hsp70s are highly conserved and exist in all organisms ranging from microorganisms to plants and humans. Structurally, Hsp70s contain a nucleotide-binding domain (NBD), acting as a ATPase domain, a substrate-binding domain (SBD), and a C-terminal lid region that covers the SBD. Usually, the NBD is around 45 kDa and the SBD is around 15 kDa. By contrast, the length or sequence of the C-terminal region varies and in some cases contains organelle-specific retention motifs [5]. The NBD and SBD are highly conserved, while the C-terminal region has varied to some extent (Figure 1).

In* Escherichia coli*, there are three Hsp70 proteins: DnaK, HscA (Hsc66), and HscC (Hsc62) [5]. Of 14 Hsp70 proteins in* Saccharomyces cerevisiae*, 9 are present in the cytosol, 3 are found in mitochondria (Ssc, Ecm, and Ssq), and 2 localize to the ER (Kar2 and Lhs1) [6]. In the unicellular alga* Chlamydomonas reinhardtii*, a total of 7 genes encode Hsp70s, with Hsp70A and Hsp70E as cytosolic proteins, Hsp70B in the chloroplast, Hsp70C and Hsp70D localize to mitochondria, and BiP1 and BiP2 are found in the ER lumen [7].* Chlamydomonas* is related to the last common ancestor of plants and animals; in contrast to the land plants that have numerous cytoplasmic Hsp70s, the alga has only one cytoplasmic Hsp70. Therefore, it seems that evolutionary and environmental selection pressure drove an increase in the types and numbers of molecular chaperones [8].

Nine cytosolic Hsp70 family members are encoded in the genome of the moss* Physcomitrella patens*, which is evolutionarily placed among the first group of plants to transition from an aquatic to a terrestrial lifestyle [9]. In addition, three chloroplast and four mitochondrial* Hsp70* genes have been identified in moss, and knockout of* cpHsp70-2* was found to be lethal [10]. The expansion of the* Hsp70* gene family was reported to result from genome and gene duplications, which are a driving force in eukaryotic evolution [11]. Rice (*Oryza sativa*) contains 32* Hsp70* genes; among these are 24 Hsp70 family and 8 Hsp110 subfamily members [12]. The* Arabidopsis Hsp70* gene family includes 18 members, 14 in the DnaK subfamily and 4 in the Hsp110/SSE subfamily. Of the DnaK subfamily proteins, six are in the cytosol, three are in chloroplast, three are in the ER, and two are in mitochondria [13]. Similarly, the* Populus trichocarpa* genome includes 20* Hsp70* genes [14]. As demonstrated by the* Hsp70* family, it appears that the complexity and function of plant genomes probably evolved after land colonization.

Studies of the* Hsp70* gene family in animals have also revealed interesting findings. In* Drosophila melanogaster*, the* Hsp70* family includes seven genes in the cytoplasmic group, but only one ER gene and one mitochondrial gene [15]. Besides that, at least 13 Hsp70 homologs are present in human, including Hsp70-5 (known as Grp78 or BiP), Hsp70-9 (known as mortalin, GRP75, or mtHsp70), and other Hsp70s in the cytosol or nucleus [16]. Based on data from fish [17], chicken [18], and buffalo [19], it appears that vertebrate animals generally have only one ER gene and that the stress-induced Hsp70s are mainly cytosolic members.

## 3. Hsp70s in Responses to Biotic and Abiotic Stress

Increasing evidences show that Hsp70s are involved in folding newly synthesized proteins, signal transduction, and protein translocation from nucleus to organelles. As housekeeping genes,* Hsp70s* are responsible for essential functions in response to various biotic and abiotic stresses.

### 3.1. Hsp70s in Prokaryotes

Hsp70, generally called DnaK in bacteria, is important for responses to several abiotic stresses. The expression of* DnaK* increases under heat, acidity, and cold stresses in* Alicyclobacillus acidoterrestris*, which is a Gram positive, rod-shaped, aerobic, acidophilic, thermophilic, and spore-forming bacterium [20]. Studies in* Staphylococcus aureus* indicated that the DnaK system contributes to heat and oxidative stress tolerance, and DnaK protein refolding machinery plays an essential role in the stress responses [21]. Hsp70/DnaK bind pathogen-associated molecular patterns (PAMPs), such as endotoxin lipopolysaccharide (LPS) and potent antimicrobial substances, which suppress pathogen proliferation [22]. Since DnaK carries out key functions to enhance shrimp resistance against the pathogen* Vibrio harveyi*, which infects aquatic animals, bacteria overproducing DnaK have been added into feed pellets to protect the white leg shrimp* Penaeus vannamei* in aquaculture [23].

### 3.2. Hsp70s in Fungi and Plants

**Figure 2:**
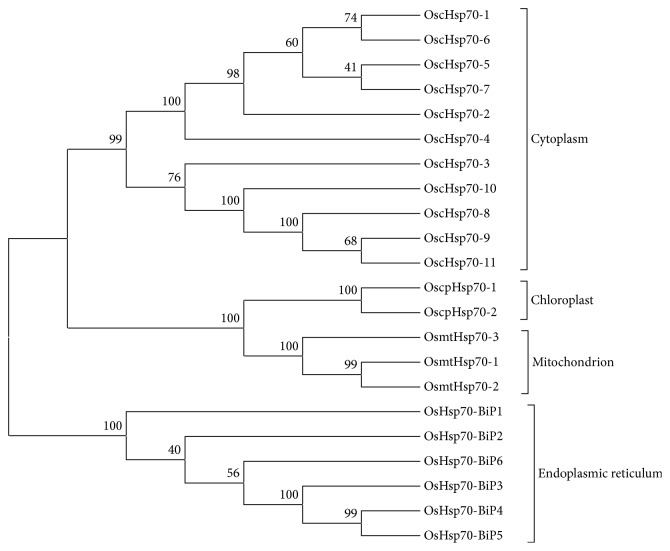
Phylogenetic tree of Hsp70 family in rice.

Hsp70s were first discovered in fruit flies in the 1960s when a lab worker accidentally increased the incubation temperature, and the basic function of these proteins is recognized to be as molecular chaperones [24]. Hsp70s are evolutionarily highly conserved. In plants, mitochondrial and plastid Hsp70s have relatively high sequence similarity to DnaK (the bacterial Hsp70 homolog), ranging from 50% to 63%, while the other Hsp70s members are more closely related to cytosolic Hsp70s [25]. The cytosolic Hsp70 family members display up to 75% amino acid identity to other plant Hsp70 proteins of this group [26]. The sequences of Hsp70 homologs from the different compartmentations have similarity and peculiarity with each other. According to those foregoing researches, ER arise from the early stages of the eukaryotic cell, while mitochondria and chloroplast are the accepted endosymbiotic origin organelles. Here we showed an example in rice (Figure 2). From the phylogenetic tree of Hsp70 family in rice, we can clearly see that the evolutionary relation of those Hsp70s is consistent with the origin of compartmentations.

#### 3.2.1. Roles of Cytosolic Hsp70s

The cytosolic Hsp70 proteins can be divided into two subgroups. One group is the heat shock cognate (Hsc) 70 proteins, which are involved in normal cellular activities such as protein folding and intracellular targeting. Another group is Hsp70s, which function in responses to various stresses and probably participate in the refolding of denatured or aggregated proteins [6].

*Saccharomyces cerevisiae* contains four classes of cytosolic Hsp70, termed Ssa, Ssb, Sse, and Ssz. The four members of Ssa in* S. cerevisiae* represent the major cytosolic Hsp70 family, and among them Ssa3 and Ssa4 confer greater resistance to H_2_O_2_ [27]. The Hsp70 Ssz1 can bind the Hsp40 Zuotin and form a heterodimeric ribosome-associated complex (known as RAC) to stimulate Ssb ATPase activity [28]. Moreover, Ssb might affect membrane protein biogenesis in response to salt [29]. Sse1 and Sse2 proteins represent the Hsp110 subfamily in* S. cerevisiae*. Loss of* Sse1* renders cells slow growing and temperature sensitive, and* Sse1* and* Sse2* constitute an essential gene pair, as inactivation of both genes is lethal [30].

As a single-celled chlorophyte green alga,* Chlamydomonas* lives in diverse environmental conditions around the world. It has been demonstrated that* Chlamydomonas Hsp70* genes are induced by light, and the expression regulation of cytosolic* Hsp70A* is mediated by a heat shock-independent pathway [31]. In maize, cytosolic Hsp70 regulates the abscisic acid- (ABA-) induced antioxidant response during drought and heat stress. Upon exposure to abiotic stresses, H_2_O_2_ production is induced and leads to enhanced activities of antioxidant enzymes, with subsequent increases in the endogenous ABA levels and Hsp70 accumulation [32].

To identify the functions of Hsp110/SSE subfamily members in* Arabidopsis*, artificial microRNA (amiRNA) technology has been used to silence* AtHsp70-14* and* AtHsp70-15* genes. The transgenic amiRNA-*AtHsp70-14/15* plants showed growth retardation and their leaves shriveled faster than those of the control. Besides, AtHsp70-15-deficient plants were more sensitive to ABA and heat treatment [33].

In short, the cytosolic Hsp70s in plants and fungi serve as key factors in responses to ROS, salt, and ABA stresses.

#### 3.2.2. Roles of ER Hsp70s

The accumulation of unfolded or misfolded proteins in the ER lumen is thought to cause ER stress and activate a protective signaling pathway known as the unfolded protein response (UPR) [34], which involves ER-resident molecular chaperone Hsp70s called Binding immunoglobulin Proteins (BiPs). The unfolded or misfolded proteins are recognized and sent out of the organelle for degradation.

Overexpression of BiP in transgenic tobacco (*Nicotiana tabacum* L. cv Havana) plants can prevent cellular dehydration, reducing the effects of drought [35]. Nevertheless, soybean BiP functions in drought tolerance and drought-induced leaf senescence [36]. Compared to wild type, the rates of photosynthesis and CO_2_ assimilation are less inhibited by drought in transgenic lines overexpressing BiP, and these lines are resistant to drought, although the mechanism is unclear [36].

In* Citrus*, the ER-resident* Hsp70-5* shows seed-specific expression and has a key function in seed desiccation tolerance. Furthermore,* Citrus Hsp70-5* is highly expressed in response to biotic stresses, such as* Xylella* infection and* Citrus tristeza* viral infection, due to activation of the UPR signaling pathway [37].

The mechanism of BiP-mediated stress resistance is not clear at all; therefore, the general mechanisms for protection of cellular components during plant cell stresses require further investigation.

#### 3.2.3. Roles of Mitochondrial Hsp70s

Most mitochondrial protein precursors are synthesized in the cytosol and subsequently imported into the mitochondrial matrix. It has been demonstrated that mtHsp70 plays a prominent role in mitochondrial biogenesis. According to previous reports, mtHsp70 binds to protein precursors as they come into the matrix and provides the driving force for the import reaction through ATP hydrolysis. After import, mtHsp70 probably participates in substrate refolding [10]. In pea (*Pisum sativum*) leaves, mtHsp70 function has been demonstrated to be related to cellular development, and the expression of* mtHsp70* is suppressed as the plant ages [38].

Studies of mtHsp70 functions in response to abiotic stress in plants are limited, although* mtHsp70* genes have been identified from many plant species. The moss* Pohlia nutans* lives in the Antarctic polar environment, and* PnHsp70*, encoding a mitochondrial chaperone, is induced by low or high temperature, salinity, drought, or PEG treatment, as well as UV radiation [39].

In general, although the functions of plant mitochondrial Hsp70 proteins under stress conditions have not been well studied, they are known to be crucial for cell survival.

#### 3.2.4. Roles of Chloroplast Hsp70s

In* Chlamydomonas*, the chloroplast-localized Hsp70B plays a key role in protection against photoinhibition. The expression of* Hsp70B* is increased not only under heat shock, but also under high light or oxidative stress conditions [40]. Hsp70s are important in pathological processes at the same time. A wheat (*Triticum aestivum* L.) chloroplast TaHsp70 plays a crucial role in stress-related responses and in defense responses elicited by infection with stripe rust fungus via a JA-dependent signal transduction pathway [41]. During* Arabidopsis* seed germination, cpHsp70-1 is important for root growth in response to the heat treatment. Analyses of* cpHsp70-1* and* cpHsp70-2* single knockout mutants indicated that cpHsp70s are essential for plant development and that the two cpHsp70s most likely have redundant but also distinct functions [42], while* cpHsp70-1* is more highly expressed in leaf than all other Hsp70s, suggesting a specific role of cpHsc70-1 in chloroplasts [43]. Similarly in rice, OsHspCP1 plays an important role in chloroplast development during heat stress [44].

In summary, the chloroplast Hsp70s carry out pivotal functions in processes related to growth and development and are regulated by environmental factors such as light, heat, and pathological stresses.

### 3.3. Hsp70s in Animals

Hsp70 represents the best-characterized class of cytoprotection proteins, and its expression is markedly induced by a variety of stresses, such as heat shock, UV irradiation, and exposure to heavy metals. However, the distribution and accumulation of Hsp70s are highly tissue specific in different development stages of animals.

In insects (e.g.,* Drosophila melanogaster*), zebrafish (*Danio rerio*), and mammals,* Hsp70* has been shown to be expressed in a stage- and tissue-specific manner. In spermatogonial cells of* D. melanogaster*, the constitutive presence of Hsp70 proteins was shown to be more stable than in other cell types during heat shock [45]. In bull spermatozoa, there is a positive correlation between* Hsp70* expression level and sperm motility [46]. Interestingly, in zebrafish embryos, in situ hybridization experiments indicated that the amount of* Hsp70* mRNA localized in the brain, eye, otic vesicle, and yolk sac was enhanced under heat stress conditions [47]. In rainbow trout, Hsp70 accumulates in juvenile trout tissues including the gill and liver upon exposure to heavy metals [48].

In pigs, the expression level of* Hsp70* in blood lymphocytes increased significantly under cold stress at −10 to −4°C, and it can serve as a molecular biomarker of cold stress in animals [49].

Among human Hsp70 family members, the ER-located Hsp70-5 and cytosolic Hsp70-8 are regarded as essential housekeeping genes [50]. Furthermore, the mitochondrial Hsp70-9 participates in several cellular processes related to maintaining protein homeostasis. In mice, overexpression of* mtHsp70* in retinal ganglion cells (RGCs) can suppress vision loss and optic atrophy. In the near future, mtHsp70 will likely emerge as a therapeutic target for optic neuritis, Parkinson's and Alzheimer's diseases, and cancers [51], whereas human Hsp70-1 and Hsp70-2 also display different functions in different cancer cells. Hsp70-1 is related to the origins of malignant tumors and may be a relevant cancer survival protein. By contrast, Hsp70-2 is abundantly expressed in the nervous system, and its expression is increased in metastatic breast cancers [52].

The human Hsp70s have attracted extensive attention worldwide, because they present a new approach for treatment of various kinds of diseases affecting mitochondria.

## 4. Conclusions

Hsp70s are widely distributed in numerous organisms from bacteria to animals. They play important roles in essential cellular processes and also in stress-related processes of the cells. In this review, we summarized their functions and application in response to a variety of stresses in bacteria, fungi, plants, and animals. Given that Hsp70 is also a potential therapeutic target for a variety of human diseases, further studies will be required to fully elucidate the roles of Hsp70s in all living species.

## Supplementary Material

The Supplementary Data summarizes the information of all Hsp70s mentioned in the text, including the gene names, genes locus, cellular localization and so on.

## References

[1] Hartl F. U., Bracher A., Hayer-Hartl M. (2011). Molecular chaperones in protein folding and proteostasis. *Nature*.

[2] Morimoto R. I. (1998). Regulation of the heat shock transcriptional response: cross talk between a family of heat shock factors, molecular chaperones, and negative regulators. *Genes & Development*.

[3] Karlin S., Brocchieri L. (1998). Heat shock protein 70 family: multiple sequence comparisons, function, and evolution. *Journal of Molecular Evolution*.

[4] Makumire S., Revaprasadu N., Shonhai A., Riggs P. D. (2015). DnaK protein alleviates toxicity induced by citrate-coated gold nanoparticles in *Escherichia coli*. *PLoS ONE*.

[5] Zhu X. T., Zhao X., Burkholder W. F. (1996). Structural analysis of substrate binding by the molecular chaperone DnaK. *Science*.

[6] Kominek J., Marszalek J., Neuvéglise C., Craig E. A., Williams B. L. (2013). The complex evolutionary dynamics of Hsp70s: a genomic and functional perspective. *Genome Biology and Evolution*.

[7] Schroda M. (2004). The *Chlamydomonas* genome reveals its secrets: chaperone genes and the potential roles of their gene products in the chloroplast. *Photosynthesis Research*.

[8] Waters E. R. (2003). Molecular adaptation and the origin of land plants. *Molecular Phylogenetics and Evolution*.

[9] Rensing S. A., Lang D., Zimmer A. D. (2008). The Physcomitrella genome reveals evolutionary insights into the conquest of land by plants. *Science*.

[10] Shi L.-X., Theg S. M. (2010). A stromal heat shock protein 70 system functions in proteinimport into chloroplasts in the moss physcomitrella patens. *Plant Cell*.

[11] Rensing S. A., Ick J., Fawcett J. A. (2007). An ancient genome duplication contributed to the abundance of metabolic genes in the moss *Physcomitrella patens*. *BMC Evolutionary Biology*.

[12] Sarkar N. K., Kundnani P., Grover A. (2013). Functional analysis of Hsp70 superfamily proteins of rice (*Oryza sativa*). *Cell Stress and Chaperones*.

[13] Lin B.-L., Wang J.-S., Liu H.-C. (2001). Genomic analysis of the Hsp70 superfamily in *Arabidopsis thaliana*. *Cell Stress and Chaperones*.

[14] Zhang J., Liu B. I., Li J. B. (2015). *Hsf* and *Hsp* gene families in *Populus*: genome-wide identification, organization and correlated expression during development and in stress responses. *BMC Genomics*.

[15] Nikolaidis N., Nei M. (2004). Concerted and nonconcerted evolution of the Hsp70 gene superfamily in two sibling species of nematodes. *Molecular Biology and Evolution*.

[16] Boswell-Casteel R. C., Johnson J. M., Duggan K. D., Tsutsui Y., Hays F. A. (2015). Overproduction and biophysical characterization of human HSP70 proteins. *Protein Expression and Purification*.

[17] Place S. P., Hofmann G. E. (2005). Constitutive expression of a stress-inducible heat shock protein gene, hsp70, in phylogenetically distant Antarctic fish. *Polar Biology*.

[18] Givisiez P. E. N., Da Silva M. M., Mazzi C. M. (2000). Heat or cold chronic stress affects organ weights and Hsp70 levels in chicken embryos. *Canadian Journal of Animal Science*.

[19] Mishra A., Hooda O. K., Singh G., Meur S. K. (2011). Influence of induced heat stress on HSP70 in buffalo lymphocytes. *Journal of Animal Physiology and Animal Nutrition*.

[20] Jiao L., Ran J., Xu X., Wang J. (2015). Heat, acid and cold stresses enhance the expression of DnaK gene in *Alicyclobacillus acidoterrestris*. *Food Research International*.

[21] Singh V. K., Utaida S., Jackson L. S., Jayaswal R. K., Wilkinson B. J., Chamberlain N. R. (2007). Role for dnaK locus in tolerance of multiple stresses in *Staphylococcus aureus*. *Microbiology*.

[22] Hu B., Phuoc L. H., Sorgeloos P., Bossier P. (2014). Bacterial HSP70 (DnaK) is an efficient immune stimulator in *Litopenaeus vannamei*. *Aquaculture*.

[23] Sinnasamy S., Mat Noordin N., MacRae T. H. (2015). Ingestion of food pellets containing *Escherichia coli* overexpressing the heat-shock protein DnaK protects *Penaeus vannamei* (Boone) against *Vibrio harveyi* (Baumann) infection. *Journal of Fish Diseases*.

[24] Gong W. J., Golic K. G. (2006). Loss of hsp70 in drosophila is pleiotropic, with effects on thermotolerance, recovery from heat shock and neurodegeneration. *Genetics*.

[25] Wimmer B., Lottspeich F., Van Der Klei I., Veenhuis M., Gietl C. (1997). The glyoxysomal and plastid molecular chaperones (70-kDa heat shock protein) of watermelon cotyledons are encoded by a single gene. *Proceedings of the National Academy of Sciences of the United States of America*.

[26] Sung D.-Y., Kaplan F., Guy C. L. (2001). Plant Hsp70 molecular chaperones: protein structure, gene family, expression and function. *Physiologia Plantarum*.

[27] Hasin N., Cusack S. A., Ali S. S., Fitzpatrick D. A., Jones G. W. (2014). Global transcript and phenotypic analysis of yeast cells expressing Ssa1, Ssa2, Ssa3 or Ssa4 as sole source of cytosolic Hsp70-Ssa chaperone activity. *BMC Genomics*.

[28] Fiaux J., Horst J., Scior A. (2010). Structural analysis of the Ribosome-associated Complex (RAC) reveals an unusual Hsp70/Hsp40 interaction. *The Journal of Biological Chemistry*.

[29] Peisker K., Chiabudini M., Rospert S. (2010). The ribosome-bound Hsp70 homolog Ssb of *Saccharomyces cerevisiae*. *Biochimica et Biophysica Acta—Molecular Cell Research*.

[30] Shaner L., Wegele H., Buchner J., Morano K. A. (2005). The yeast Hsp110 Sse1 functionally interacts with the Hsp70 chaperones SSa and Ssb. *The Journal of Biological Chemistry*.

[31] Kropat J., von Gromoff E. D., Müller F. W., Beck C. F. (1995). Heat shock and light activation of a *Chlamydomonas* HSP70 gene are mediated by independent regulatory pathways. *Molecular & General Genetics*.

[32] Hu X., Liu R., Li Y. (2010). Heat shock protein 70 regulates the abscisic acid-induced antioxidant response of maize to combined drought and heat stress. *Plant Growth Regulation*.

[33] Jungkunz I., Link K., Vogel F., Voll L. M., Sonnewald S., Sonnewald U. (2011). AtHsp70-15-deficient Arabidopsis plants are characterized by reduced growth, a constitutive cytosolic protein response and enhanced resistance to TuMV. *Plant Journal*.

[34] Kleizen B., Braakman I. (2004). Protein folding and quality control in the endoplasmic reticulum. *Current Opinion in Cell Biology*.

[35] Alvim F. C., Carolino S. M. B., Cascardo J. C. M. (2001). Enhanced accumulation of BiP in transgenic plants confers tolerance to water stress. *Plant Physiology*.

[36] Valente M. A. S., Faria J. A. Q. A., Soares-Ramos J. R. L. (2009). The ER luminal binding protein (BiP) mediates an increase in drought tolerance in soybean and delays drought-induced leaf senescence in soybean and tobacco. *Journal of Experimental Botany*.

[37] Fietto L. G., Costa M. D. L., Cruz C. D., Souza A. A., Machado M. A., Fontes E. P. B. (2007). Identification and in silico analysis of the *Citrus* HSP70 molecular chaperone gene family. *Genetics and Molecular Biology*.

[38] Dudley P., Wood C. K., Pratt J. R., Moore A. L. (1997). Developmental regulation of the plant mitochondrial matrix located HSP70 chaperone and its role in protein import. *FEBS Letters*.

[39] Liu S., Wang J., Cong B., Huang X., Chen K., Zhang P. (2014). Characterization and expression analysis of a mitochondrial heat-shock protein 70 gene from the Antarctic moss Pohlia nutans. *Polar Biology*.

[40] Schroda M., Vallon O., Wollman F.-A., Beck C. F. (1999). A chloroplast-targeted heat shock protein 70 (HSP70) contributes to the photoprotection and repair of photosystem II during and after photoinhibition. *Plant Cell*.

[41] Duan Y.-H., Guo J., Ding K. (2011). Characterization of a wheat HSP70 gene and its expression in response to stripe rust infection and abiotic stresses. *Molecular Biology Reports*.

[42] Su P.-H., Li H.-M. (2008). Arabidopsis stromal 70-kD heat shock proteins are essential for plant development and important for thermotolerance of germinating seeds. *Plant Physiology*.

[43] Sung D. Y., Vierling E., Guy C. L. (2001). Comprehensive expression profile analysis of the *Arabidopsis* hsp70 gene family. *Plant Physiology*.

[44] Kim S.-R., An G. (2013). Rice chloroplast-localized heat shock protein 70, OsHsp70CP1, is essential for chloroplast development under high-temperature conditions. *Journal of Plant Physiology*.

[45] Lakhotia S. C., Prasanth K. V. (2002). Tissue- and development-specific induction and turnover of hsp70 transcripts from loci 87A and 87C after heat shock and during recovery in *Drosophila melanogaster*. *Journal of Experimental Biology*.

[46] Zhang X. G., Hong J. Y., Yan G. J., Wang Y., Li Q., Hu J. (2015). Association of heat shock protein 70 with motility of frozen-thawed sperm in bulls. *Czech Journal of Animal Science*.

[47] Yamashita M., Uchino K., Taguchi Y. (2003). Stress response and apoptosis in zebrafish embryos. *Aquatic Genomic: Steps toward a Great Future*.

[48] Misra S., Zafarullah M., Price-Haughey J., Gedamu L. (1989). Analysis of stress-induced gene expression in fish cell lines exposed to heavy metals and heat shock. *Biochimica et Biophysica Acta—Gene Structure and Expression*.

[49] Ji H., Wu Y. K., Guo J. R. (2012). Effects of cold stress on expression of Hsp70 gene in blood lymphocyte of piglets. *Journal of Animal and Veterinary Advances*.

[50] Daugaard M., Rohde M., Jäättelä M. (2007). The heat shock protein 70 family: highly homologous proteins with overlapping and distinct functions. *FEBS Letters*.

[51] Dores-Silva P. R., Barbosa L. R. S., Ramos C. H. I., Borges J. C. (2015). Human mitochondrial Hsp70 (mortalin): shedding light on ATPase activity, interaction with adenosine nucleotides, solution structure and domain organization. *PLoS ONE*.

[52] Mosser D. D., Morimoto R. I. (2004). Molecular chaperones and the stress of oncogenesis. *Oncogene*.

